# A practical spatial analysis method for elucidating the biological mechanisms of cancers with abdominal dissemination in vivo

**DOI:** 10.1038/s41598-022-24827-w

**Published:** 2022-11-24

**Authors:** Yukihide Ota, Shinya Sato, Mitsuyo Yoshihara, Yoshiyasu Nakamura, Etsuko Miyagi, Yohei Miyagi

**Affiliations:** 1 Molecular Pathology and Genetics Division, Kanagawa Cancer Center Research Institute, Yokohama, Kanagawa 241-8515 Japan; 2 Morphological Information Analysis Laboratory, Kanagawa Cancer Center Research Institute, Yokohama, Kanagawa 241-8515 Japan; 3 Department of Obstetrics and Gynecology, Yokohama City University Graduate School of Medicine, Yokohama, Kanagawa 236-0004 Japan; 4 Department of Pathology, Kanagawa Cancer Center, 2-3-2 Nakao, Asahi Ward, Yokohama, Kanagawa 241-8515 Japan

**Keywords:** Cancer microenvironment, Cancer models

## Abstract

Elucidation of spatial interactions between cancer and host cells is important for the development of new therapies against disseminated cancers. The aim of this study is to establish easy and useful method for elucidating spatial interactions. In this study, we developed a practical spatial analysis method using a gel-based embedding system and applied it to a murine model of cancer dissemination. After euthanization, every abdominal organ enclosed in the peritoneum was extracted en bloc. We injected agarose gel into the peritoneal cavities to preserve the spatial locations of the organs, including their metastatic niches, and then produced specimens when the gel had solidified. Preservation of the original spatial localization was confirmed by correlating magnetic resonance imaging results with the sectioned specimens. We examined the effects of spatial localization on cancer hypoxia using immunohistochemical hypoxia markers. Finally, we identified the mRNA expression of the specimens and demonstrated the applicability of spatial genetic analysis. In conclusion, we established a practical method for the in vivo investigation of spatial location-specific biological mechanisms in disseminated cancers. Our method can elucidate dissemination mechanisms, find therapeutic targets, and evaluate cancer therapeutic effects.

## Introduction

Several biological mechanisms, including those involving epigenetically regulated signal transductions, occur only in specific spatial locations^1–4^. In cancer research, spatial cell–cell interactions are important at every step of cancer progression including metastasis and dissemination. For example, spatial transcriptome analysis has shown that the lymphoid areas close to the tumor region specifically expresses cancer-promoting genes when compared to the lymphoid areas more distant from the tumor area^4^. Moreover, spatial tissue analysis has shown that cancer cells promote remodeling of the tumor microenvironment via transforming growth factor beta signaling^5^. Therefore, spatial analysis is essential for understanding the precise interactions between cancer cells and their surrounding host cells especially in the metastasized or disseminated microenvironments.

Spatial information-based analysis requires tools that provide detailed anatomical information. Currently, spatial information is obtained using imaging techniques such as computed tomography, magnetic resonance imaging (MRI), and ultrasound scanning. These imaging systems are used to assess the extent of tumor spread and metastasis for clinical staging and treatment selection^6–8^. Although these tools provide relevant spatial information, when spatial location-specific biological mechanisms in the tumor microenvironment are examined in detail, other tools that can directly access tumor tissues are required. More recently, sophisticated in vivo imaging methodologies have been developed, such as tissue-clearing technology-based 3D imaging^9,10^ and cryo-fluorescence tomography^11,12^. These methods provide very detailed images, preserving the original localization of cells and extracellular matrix in organs, tissues, and cells in 3D images; however, these technologies have limitations. Because the former technology is mainly employed for detecting endogenous fluorescent signals and are not suitable for analyzing the expression of multiple genes/proteins. Also, the latter technology is employed for analyzing cells and tissues in the order of micrometers and is not suitable for observing organ-to-organ interactions at the millimeter or centimeter scale. Therefore, new methods to analyze 2D organ-to-organ interactions while preserving the original spatial information and to access the expression of multiple proteins/RNAs are required to investigate tumor microenvironment between different organs.

Pathological specimens possess spatial information as well as biological information. Effective genetic spatial analysis tools have recently become commercially available and are frequently applied to histopathological specimens^13–15^. In general, histopathological analysis requires the extraction and dissection of each organ separately without preserving any spatial information. Although some methods of preserving spatial information have been proposed, they are inadequate for the preservation of precise spatial data^16,17^.

Herein, we developed a novel and simple method for preparing specimens intended for spatial biological investigations in mice; the method involves solidifying all the abdominal organs together using agarose gel. By focusing on disseminated ovarian cancer, we demonstrated the utility of our system by using it to investigate two spatial location-dependent biological mechanisms—hypoxia and lipid metabolism. We also showed that this method preserves RNA, thereby demonstrating its applicability to spatial genetic and epigenetic analyses. Our model provides complex spatial information including metastatic niches. Our model can reveal the previously undetectable biological mechanisms of the microenvironments and meet the needs of any research that requires in vivo spatial information, such as elucidation of dissemination mechanisms, investigation of new therapeutic targets, and evaluation of cancer therapeutic effects.

## Results

### Establishment of the gel embedding method that preserves spatial information

**Figure 1:**
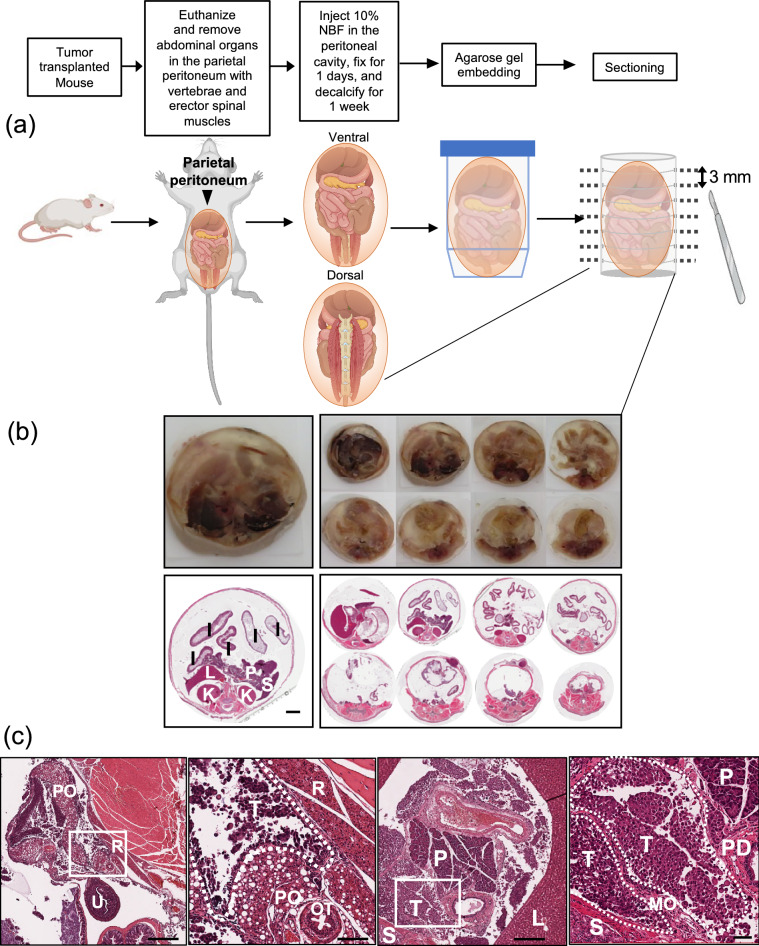
Spatial sectioning method and representative sectioned images. (**a**) Spatial sectioning procedure. (**b**) Preservation of spatial information pertaining to the sectioned specimens. Sectioned organs (top) and macro images of hematoxylin and eosin (H&E)-stained sections (bottom) from nude BALB/c mice with ES-2 ovarian cancer dissemination are shown. *I* Intestine, *K* kidney, *L* liver, *P* pancreas, *S* spleen. Scale bar: 2 mm. (**c**) Preserved metastatic niches composed of multiple organs/tissues. *T* disseminated cancer, *U* uterus, *R* retroperitoneal adipose tissue, *PO* periovarian adipose tissue, *OT* ovarian tube, *L* liver, *P* pancreas, *PD* pancreatic duct, *MO* major omentum, *S* stomach. Scale bar from left to right images: 500 µm, 100 µm, 500 µm, and 100 µm, respectively.

To develop a multiorgan evaluation system that preserves spatial information, we employed an agarose gel-embedding method. We applied this system to a murine model of ovarian cancer dissemination because of the importance of three-dimensional interactions in cancer dissemination and therapeutic resistance^18,19^. We injected well-known ovarian cancer cell lines, ES-2 and RMG-1 into the abdomens of mice to create an ovarian dissemination model^20,21^. Except for the experiments presented in the supplementary information, we used ES-2 owing to its rapid growth and massive dissemination phenotype observed in preliminary studies. After 2–5 weeks, the cancer had spread, and ascites had developed. The organs in the parietal peritoneum were solidified with 10% agarose gel after fixation and decalcification (Fig. 1a). The spatial location of each solidified organ was thereby preserved, facilitating equidistant serial sectioning. As shown in Fig. 1b, multiple organs were observed in their original locations in each section. In the represented slide, the intestines (I), kidneys (K), pancreas (P), liver (L), and spleen (S) are visible in the same section (Fig. 1b). In addition, as shown in Fig. 1c, complicated metastatic niches were preserved in the specimens. In the pelvic peritoneal cavity, a metastatic niche formed by disseminated cancer cells, retroperitoneal adipose tissues, and periovarian adipose tissues was observed (Fig. 1c, left two images). In addition, in the upper peritoneal cavity, another metastatic niche formed by disseminated cancer cells, pancreatic tissue, the pancreatic duct, the major omentum, and the stomach was observed (Fig. 1c, right two images). These results demonstrated that we successfully established an in vivo specimen preparation method for spatial analysis.

### Evaluation of the accuracy of our system for preserving spatial information by MRI

**Figure 2:**
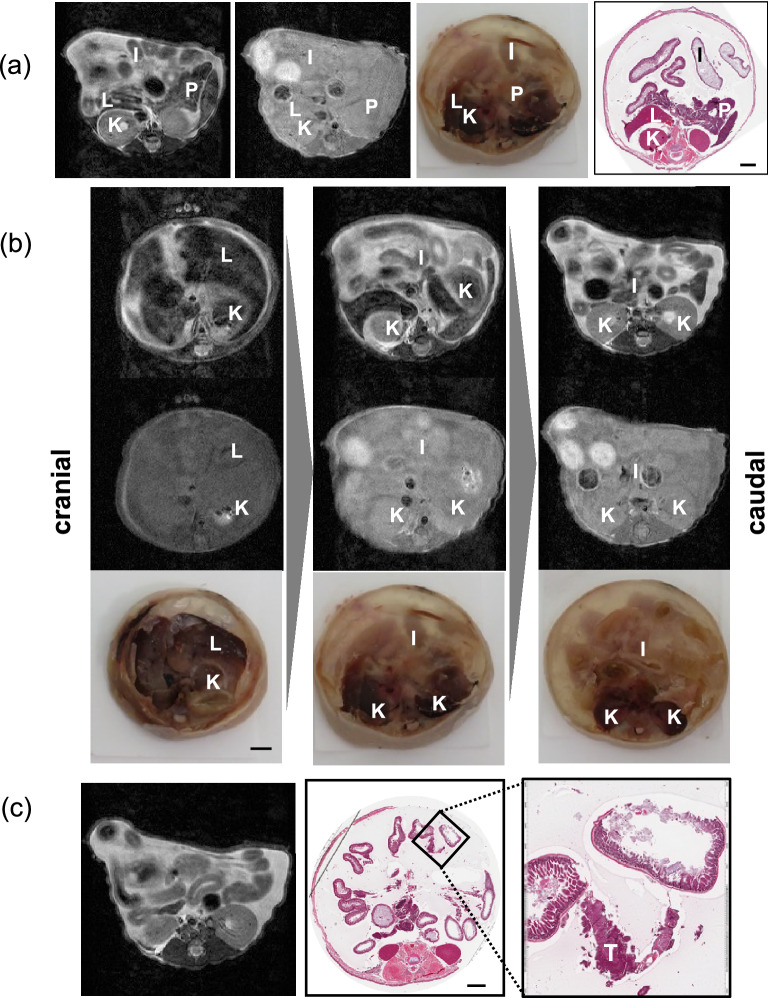
(**a**) Preservation of spatial information in the sectioned slides. Magnetic resonance imaging (MRI) images [left: T2-weighted image (T2WI); right: T1-weighted image (T1WI)], sectioned organs, and macro images of hematoxylin and eosin (H&E)-stained section specimens from nude BALB/c mice with ES-2 ovarian cancer dissemination are shown. *I* Intestine, *K* kidney, *L* liver, *P* pancreas, *S* spleen. Scale bar: 2 mm. (**b**) Sequential MRI images [upper images: T2-weighted images (T2WIs); lower images: T1-weighted images (T1WIs)] and sectioned organs at the same anatomical level as the MRI images. Scale bar: 2 mm. (**c**) Comparison between the spatial information obtained using MRI and that obtained from the sectioned slides created using our method. A disseminated small nodule (inset, T) was visible in the sectioned slide. Scale bar: 2 mm.

To evaluate the accuracy and reproducibility of our system for obtaining abdominal spatial information, we applied MRI to our dissemination model. We imaged the disseminated cancer in the model mice and prepared sections from the mice after imaging. Figure 2a,b showed the MRI images of disseminated mouse and sectioned organs of the same mouse at the same anatomical level with the MRI images. Using our method, the positions of the solid organs as determined using MRI, were preserved in the macro specimens and in the H&E-stained sections. Meanwhile, the hollow organs were mobile in the peritoneal cavity, therefore the positions of these organs were changed (Fig. 2a,b). Sequential horizontal images of the mouse obtained using MRI confirmed that the spatial information of the solid organs was preserved throughout the peritoneal cavity in the sectioned tissues (Fig. 2b). Therefore, MRI confirmed the reproducibility of the spatial information obtained using our system. Histopathological analysis also allowed us to detect very small tumor nodules that evaded detection with MRI (Fig. 2c). Thus, our model allows for a detailed analysis of lesions while preserving spatial information.

### Functional analysis: preserving spatial information pertaining to hypoxia in the tumor environment

**Figure 3:**
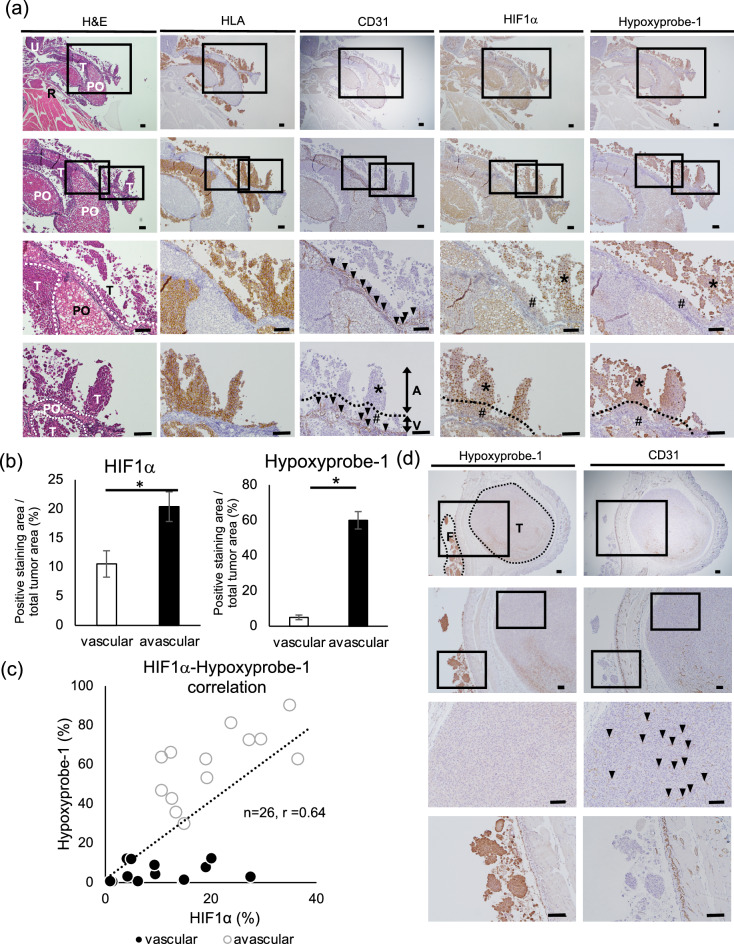
Expression analysis of hypoxia-related proteins in ES-2-disseminated cancer using our method. (**a**) Images of hematoxylin and eosin (H&E)-, HLA-, CD31-, HIF1α-, and hypoxyprobe-1-stained tissue. “*” and “A” indicates an avascular cancer region; “#” and “V” indicates a vascular cancer region; arrowheads indicate CD31-positive blood vessels. The dotted line indicates the border between vascular and avascular region. *T* disseminated cancer, *U* uterus, *R* retroperitoneal adipose tissue, *PO* periovarian adipose tissue. Scale bars: 100 µm. (**b**) Statistical analysis of HIF1α- and hypoxyprobe-1-stained tumor areas in the vascular regions (i.e., tumor areas within 100 µm of CD31-positive vessels) and avascular regions (i.e., tumor areas 100 µm away from CD31-positive vessels); n = 26. An unpaired *t*-test was performed for statistical analysis. *P < 0.05. (**c**) Correlation coefficient between HIF1α and hypoxyprobe-1; n = 26; *R* = 0.64. (**d**) hypoxyprobe-1 (left) and CD31 (right) staining for peritoneal and subcutaneous cancer tissue. The arrowheads indicate CD31-positive blood vessels. *T* disseminated cancer, *F* cancer cells floating in ascitic fluid. Scale bars: 100 µm.

**Figure 4:**
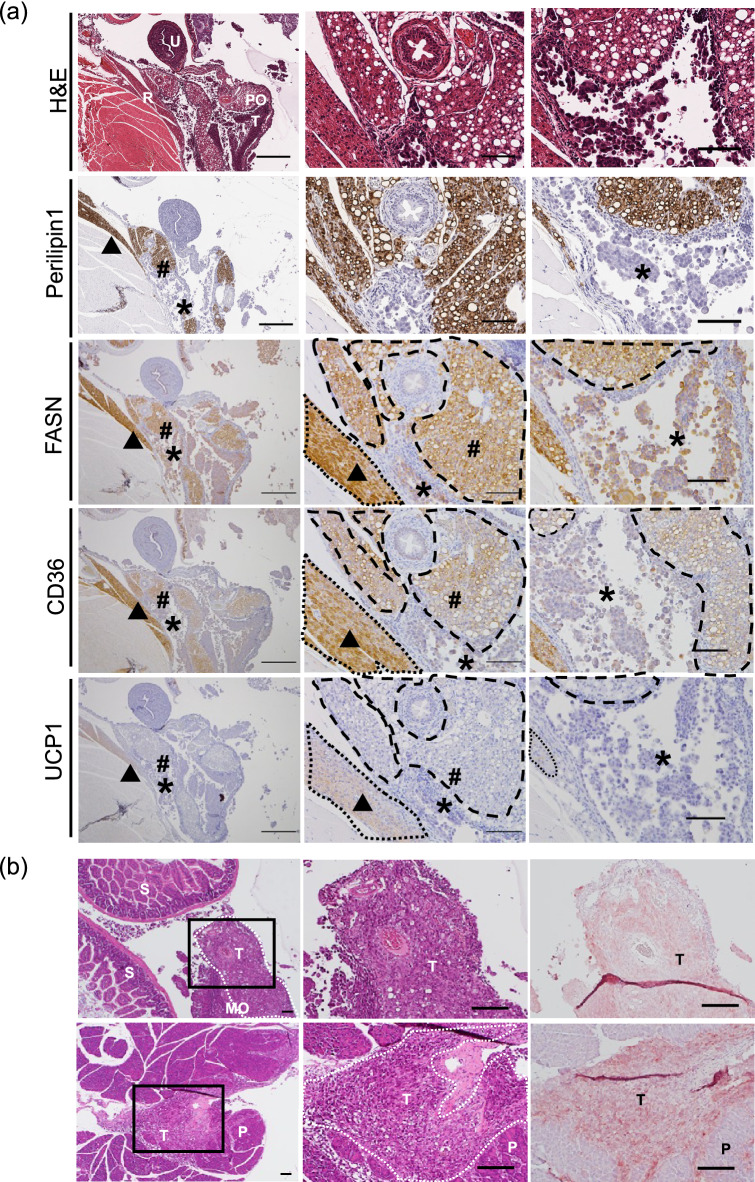
(**a**) Expression of lipid metabolism-related molecules. FASN, CD36, and UCP1 were stained for ES-2-disseminated cancer cells, visceral white adipocytes, and retroperitoneal brown adipocytes. “*” indicates disseminated or floating cancer cells; “#” indicates visceral adipocytes; “▲” indicates brown adipocytes. The dotted lines indicate brown adipose tissue. The dashed lines indicate white/visceral adipose tissue. *T* disseminated cancer, *U* uterus, *R* retroperitoneal adipose tissue, *PO* periovarian adipose tissue. Scale bar: 2 mm (left), 500 µm (middle), and 100 µm (right). (b) Staining with hematoxylin and eosin (H&E) (left, middle) and RNA-ISH (right) for disseminated human cancer cells using an anti-human mRNA-positive control probe. *T* disseminated cancer, *S* small intestine, *MO* major omentum, *P* pancreas. Scale bar: 500 µm.

Next, we attempted to determine the potential usefulness of our method for elucidating biological functions in a metastatic niche only specifically observed using our method. In general, although severe hypoxia is associated with metastasized cancer, the exact extent of the hypoxia is unclear^22^. Determining the degree of hypoxia associated with disseminated cancers may facilitate the development of effective therapies. We performed IHC analysis of HIF1α and hypoxyprobe-1 (pimonidazole derivatives), which are hypoxia markers^23,24^, to quantify the hypoxia associated with the cancer cells. As shown in Fig. 3a, we measured hypoxia in the metastatic niche shown in Fig. 1, which was composed of disseminated cancer cells, the retroperitoneal connective tissue, the uterus, and periovarian visceral adipose tissue. It was possible to visualize the disseminated ovarian cancer cells using anti-HLA staining. We compared the extent of HIF1α and hypoxyprobe-1 staining in the vascular and avascular areas. Both HIF1α and hypoxyprobe-1 staining was significantly more prevalent in the avascular regions than in the vascular regions (Fig. 3b). There was a significant correlation between the staining patterns of HIF1α and hypoxyprobe-1 in the same cases (Fig. 3c). Interestingly, there was a greater degree of hypoxyprobe-1 staining in the cancer cells floating in the peritoneal cavity than in the intra-abdominal cancer cells supplied by numerous CD31-positive vessels (Fig. 3a,d). These results suggest that our model can be used to accurately assess the level of hypoxia in spatially disseminated abdominal tumors. Next, we attempted to determine metastatic niche-specific metabolic processes. Lipid metabolism is facilitated by the hypoxic conditions in cancer cells^25,26^. Furthermore, in the peritoneal cavity environment, visceral adipocytes, which provide lipid droplets, are major host cells that interact with disseminated cancer^27,28^. We used FASN staining to investigate lipid metabolism in the cancer cells and found that the disseminated cancer cells stained positive for FASN (Fig. 4a) in the metastatic niche, as in Fig. 3. CD36 is a key molecule in fatty acid transport and is generally expressed in both white and brown adipocytes; the disseminated cancer cells were stained slightly for CD36 (Fig. 4a). Moreover, it has been reported that white adipocytes positively support tumor development^29,30^. In accordance with those reports, the disseminated cancer cells mainly attached to and infiltrated UCP1 (a marker for brown adipocytes) negative and Perillipin1 (a marker for pan-adipocytes) positive white adipocytes in the metastatic niche (Fig. 4a).

### Potential analysis of spatial gene expression

Finally, we assessed the applicability of our system to the latest spatial genetic analysis techniques^15,31^ by performing RNA-ISH on the specimens. As indicated in red in Fig. 4b, there was significant human mRNA expression in the disseminated human cancer cells (red colored area) that infiltrated the greater omentum and pancreas in the metastatic niche. Moreover, the specific mRNA expression of a lipid transport-specific gene (FABP4) was also observed in the retroperitoneal adipose tissue (Supplementary Fig. S2). These results suggest that spatial mRNA expression analysis can be performed on specimens using our method.

## Discussion

We established a novel method for comprehensively accessing detailed spatial information pertaining to biological mechanisms in the peritoneum. Our method can be used to evaluate the volume of disseminated tumors and investigate spatial location-specific cancer development mechanisms. To date, evaluation of the volume of disseminated tumors has mainly been based on the macroscopic examination of resected specimens or in vivo fluorescence imaging^32–34^. Our method offers definite advantages over those two techniques; compared to macroscopic examination, our approach enables screening of the entire peritoneal cavity without overlooking small tumor nodules and does not require consideration of attenuation or irregular fluorescence due to the imaging conditions. Furthermore, only our method enables the examination of inter-multiple organ niche-specific biological mechanisms. Disseminated tumor masses frequently interact with multiple organs. Thus, our gel-embedding method—which preserves spatial information pertaining to multiple organs—can be used to unravel the niche-specific mechanisms of disseminated cancer.

We evaluated the accuracy of the spatial information obtained using our method by MRI. As expected, the position of every organ according to the MRI corresponded to that observed in the sectioned tissue processed using our method, which preserved the spatial information pertaining to all the abdominal organs. However, our method involves filling the peritoneal cavity with agarose gel, which changes the positions of the greater omentum and the cancer cells floating in the ascitic fluid. Therefore, our method is most suited to the evaluation of the spatial location-specific biological mechanisms of organs that are fixed to the parietal peritoneum.

Figures 3 and 4 demonstrate the potential of our method as a tool for the examination of biological mechanisms. In this regard, the oxygen concentration in cancer cells has been previously investigated^35,36^. However, the role of hypoxia in disseminated intraperitoneal cancer remains unclear. In the present study, we divided the tumors into vascular and avascular regions based on a previous report^37^. We confirmed the hypoxic status of the tissues using two major hypoxia markers, HIF1α and hypoxyprobe-1. Under hypoxic conditions, HIF1α is an important transcription factor, and its stability is regulated by the oxygen-dependent prolyl hydroxylase domain protein and von Hippel–Lindau tumor suppressor protein axis^38,39^. Pimonidazole, which is a 2-nitroimidazole adduct, has often been used as a sensitive hypoxic marker since Varghese et al. reported its use in hypoxic cells, both in vitro and in vivo^36,40^. Hypoxyprobe-1 (pimonidazole hydrochloride) can detect the adducts formed under hypoxic conditions (pO_2_ < 10 mmHg) and has been widely used, both in vitro and in vivo^41–43^. As shown in Fig. 3, the staining intensities for both HIF1α and hypoxyprobe-1 were higher in the avascular region than in the vascular region. In addition, confirming previous reports^44^, the staining area for hypoxyprobe-1, which reflects “chronic hypoxia”, was smaller than that for HIF1α in the avascular area. The cancer cells floating in ascitic fluid expressed hypoxyprobe-1 more strongly than disseminated cells. These results suggest that our model can elucidate the hypoxic conditions of disseminated cancer in metastatic niches. Lipid metabolism in cancer cells is closely related to hypoxia^25,26^. Free fatty acids act as nutrients for cancer cells under hypoxic conditions, and white adipocytes continuously release fatty acids via transporters. As shown in Fig. 4, using IHC, we investigated lipid metabolism and fatty acid intake in the metastatic niche composed of retroperitoneal connective tissue, including brown adipose tissue, the uterus, and periovarian visceral adipose tissue. The cancer cells disseminated in these white adipose tissues express FASN, which is a marker of lipid metabolism, and CD36, which is a transporter of extrinsic fatty acids. Our results suggest that, in addition to hypoxia, adipose tissue may favor disseminated/metastasized cancer cells in the peritoneal cavity. We could show that our method is able to examine spatial location-specific protein expression in a metastatic niche composed of multiple organs that has not been previously detected using other methods.

Finally, we confirmed the applicability of our method to spatial genetic expression analysis. Recently, several novel methods have been developed for spatial genetic analysis^15,31,45^. We performed RNA-ISH on the specimens, thereby demonstrating that our method preserves the mRNA in disseminated cancer in multiple organs and is appropriate for spatial gene expression analysis.

Although cancer dissemination is difficult to quantify, attempts have been made to evaluate tumor masses disseminated in the peritoneal cavity^17,46,47^. The peritoneal cancer index (PCI) method is the most frequently used technique for assessing cancer dissemination^46,48,49^. To evaluate cancer dissemination, the PCI method involves categorization of the abdominal area into nine sections and counting of visually confirmed disseminated nodules in each of those sections (Supplementary Fig. S3). In contrast, our method simultaneously evaluates the whole peritoneal cavity and the peritoneum, using IHC staining for human-derived tumor-specific proteins, such as HLA (Supplementary Fig. S3). The PCI method and our method offer different advantages and disadvantages. Compared with the PCI method, our approach preserves spatial information and may evaluate more precise and objective tumor area. However, our method has several limitations. The intraperitoneal organs solidified in agarose gel must be sliced into sections that are at least 3-mm thick. Therefore, depending on the thickness of the slice, some organs or lesions may be overlooked. Especially, the size of the disseminated tumor masses on the peritoneum was usually small less than 3 mm; thus, usual sectioning using our methods may cause missing disseminated nodules. To avoid missing small nodules, making serial step sections from one FFPE block is useful. We confirmed that the size and number of disseminated nodules were changed by the step sectioning (data not shown). Therefore, performing step sectioning in intervals of 100 µm or 200 µm from one FFPE block may provide more precise results for detecting the number and area of disseminated tumor masses. Nevertheless, our method still offers advantages for the quantitative analysis of cancer dissemination compared to the PCI method, which reportedly yields differences between the scores derived from the traditional macroscopic PCI and the pathologically evaluated PCI^50^. Another limitation of the present study is that we used a decalcifying reagent (EDTA) in addition to formalin to prepare the specimens. Such reagents reduce the sustainability of proteins and nucleic acids compared to that in fresh tissue. To assess the effects of EDTA decalcification and gel solidification on the quality of IHC, we performed IHC using specimens prepared under three different conditions (our method (EDTA+, gel+), our method without decalcification by removing vertebra (EDTA−, gel+), and the conventional method without using EDTA and gel). We performed CD31 and perillipin1 staining, and the staining intensity and staining proportion of the internal control (blood vessels and adipocytes) were not changed among these specimens (Supplementary Fig. S4). Furthermore, to assess the effect of EDTA decalcification and gel solidification on the quality of RNA, we compared the quality check of RNA among specimens prepared under the three different conditions using bioanalyzer 2100. DV200, the validation parameter of RNA quality (30% is the minimum requirement for RNA-seq analysis), were over 50% for all three RNA samples extracted from specimens prepared under different conditions (Supplementary Fig. S4). Taken together, specimens prepared using our method showed a comparable staining response and RNA quality to non-decalcified/non-gel solidified FFPE specimens and can be used to perform IHC and RNA-seq analyses as with conventional FFPE specimens. Our method requires further improvement for the detailed investigation of proteins and genetic analyses, such as the sectioning of frozen non-fixed and non-decalcified samples.

In conclusion, we established a practical method that preserves the spatial information of metastatic niches pertaining to multiple organs. We also demonstrated the usefulness of our method for investigating hypoxic conditions and lipid metabolism and indicated its potential for spatial genetic analysis. Our method is suitable for investigation of the biological mechanisms involved in spatial cell–cell, tissue–tissue, and organ–organ interactions in the peritoneal cavity. Our method can apply to elucidate dissemination mechanisms, find therapeutic targets, and evaluate cancer therapeutic effects in the future.

## Materials and methods

### Cell lines and cell culture

ES-2 (CRL-1978) was purchased from ATCC (Manassas, VA, USA), and RMG-1 (JCRB0172) was purchased from JCRB Cell Bank (Osaka, Japan). These cell lines are derived from human ovarian clear cell carcinoma and are often used in mouse xenograft models^51,52^. Cells were maintained in Roswell Park Memorial Institute 1640 medium supplemented with 10% fetal bovine serum and penicillin/streptomycin at 37 °C in a humidified atmosphere comprising 5% CO_2_. Semi-confluent cells were dissociated using 0.05% trypsin/0.02% ethylenediamine tetraacetic acid (EDTA). Centrifugation was performed for 3 min at 180 × *g* at room temperature (24–26 °C) to obtain a cell pellet.

### Cancer dissemination model

All animal studies followed the ARRIVE guidelines and were approved by the Institutional Animal Experiment Committee of Kanagawa Cancer Center, Japan (Approval No. 01-07). All methods including study design, sample size, inclusion and exclusion criteria, outcome measures, method of euthanization, tissue collection timing post-euthanasia, statistical methods, were performed in accordance with the relevant guidelines and regulations. Female BALB/c-nu/nu mice aged 6–8 weeks (Charles River Japan, Yokohama, Japan) were used for the experiments. All mice were acclimated for 2 weeks. Mice were housed in a temperature-controlled environment with a 12 h light–dark cycle and ad libitum water and diet.

We injected either 1 × 10^6^ ES-2 cells or 1 × 10^7^ RMG-1 cells intraperitoneally. We monitored the mice every day and decided the timing of sacrifice when storage of ascitic fluid was started without losing their body weights, activity, food intake, and water intake. The tumor-injected mice were sacrificed by cervical dislocation following anesthesia after 2 weeks (ES-2) or 5 weeks (RMG-1). Except for the experiments presented in the supplementary information, we used ES-2 in the present study because of its rapid growth and massive dissemination phenotype. For the hypoxia study, a HypoxyprobeTM-1 solution (Hypoxyprobe Inc., Burlington, MA, USA) in saline was injected intraperitoneally into the mice at a dosage of 60 mg/kg, 30 min before sacrifice.

### Spatial sectioning method

The workflow for preparing the specimens is shown in Fig. 1a. Disseminated cancer with some ascites developed either 2 weeks (in the case of the ES-2 cells) or 5 weeks (in the case of the RMG-1 cells) after injecting the cancer cells into the peritoneal cavities of the mice. The mice were euthanized under general anesthesia at that time-point. The abdominal organs enclosed in the parietal peritoneum—including the erector spinae muscles, the vertebrae, and the skin—were extracted en bloc. Then, 1–2 mL of 10% neutral buffered formalin was injected into each peritoneal cavity. Thereafter, the organs in the parietal peritoneum were further soaked in 10% neutral buffered formalin for 1 day in a plastic tube. Subsequently, the 10% neutral buffered formalin in both the peritoneal cavity and the tube was replaced with neutral EDTA to decalcify the vertebrae. After 1 week, the EDTA in the peritoneal cavity was replaced with 1–2 mL of 3–10% agarose gel (#E-3120-500 Gene Pure LE, ISC BioExpress, Kaysville, UT, USA), and the organs in the parietal peritoneum were soaked in a 50 mL tube filled with 10% agarose gel. The tube was stored at room temperature (24–26 °C) until the gel hardened. The organs in the parietal peritoneum were carefully removed from the tube and serially sliced into uniform 3-mm-thick sections. The sliced organs were then embedded in paraffin and sectioned for histopathological analysis. We also utilized another method that involved extracting abdominal organs en bloc without the parietal peritoneum (Supplementary Fig. S1).

### Hematoxylin and eosin staining and immunohistochemistry

Each formalin-fixed paraffin-embedded tissue specimen (4-μm thick) was placed on a glass slide and stained with hematoxylin and eosin (H&E). The deparaffinized and rehydrated slides were immersed in 0.01 M citrate at pH 6.0 (Sigma-Aldrich, St. Louis, MO, USA), and heat-induced antigen retrieval was performed in an autoclave at 110 °C for 15 min. The slides were placed on the tabletop to cool to room temperature, washed in phosphate-buffered saline, and immersed in 3% H_2_O_2_ diluted with methanol. Background Sniper (Biocare Medical, Pacheco, CA, USA) was used for blocking as required. Primary antibodies against hypoxia-inducible factor-α (HIF1α) (EP1215Y, diluted at 1:100, rabbit monoclonal IgG; Abcam, Cambridge, UK), hypoxyprobe-1 (PAb2627AP, diluted at 1: 250, rabbit polyclonal IgG antibody; Hypoxyprobe Inc., Burlington, MA, USA), CD31 (D8V9E, diluted at 1:100, rabbit monoclonal IgG; Cell Signaling Technology, Danvers, MA, USA), UCP1 (ab234430, diluted at 1:100, rabbit monoclonal IgG; Abcam), FABP4 (ab92501, diluted at 1:100, rabbit monoclonal IgG; Abcam), CD36 (ab252923, diluted at 1:100, rabbit monoclonal IgG; Abcam), fatty acid synthase (FASN; 3180S, diluted at 1:100, rabbit monoclonal IgG; Cell Signaling Technology), and human leukocyte antigen (HLA; ab52922, diluted at 1:250, rabbit monoclonal IgG; Abcam) were used for immunohistochemistry (IHC) in the present study. Histofine^®^ high stain PO (MULTI) (Nichirei, Tokyo, Japan) or Histofine^®^ simple stain mouse MAX-PO (R) (Nichirei) and a Histofine^®^ DAB substrate kit (Nichirei) were used to detect the labeled antigens. Non-specific mouse or rabbit IgG was used as a negative control. We used ImageJ software (version 1.52a)^53^ to analyze the IHC slides.

### RNA-in situ hybridization

We performed RNA-in situ hybridization (RNA-ISH) on the formalin-fixed paraffin-embedded slides. An RNAscope™ kit (Advanced Cell Diagnostics, Hayward, CA, USA) was used for RNA-ISH. RNA-ISH was performed according to manufacturer’s protocol. mRNA expression in the specimens was detected using a negative control probe, positive control probe and anti-FABP4 probe (310043, 313901 and 470641-C2; Advanced Cell Diagnostics).

### Extraction and quality check of RNA from specimens

Total RNA was extracted from specimens using an RNeasy FFPE kit (73504, QIAGEN, Hilden, Germany) following the manufacturer's protocol. Subsequently, the concentration of total RNA was measured using Nanodrop One (Thermo scientific, Rockford, CA, USA). Quality check using DV200, the validation parameter of RNA quality based on fragment size distribution^54,55^, was performed using bioanalyzer 2100 (Agilent, Santa Clara, CA, USA) following the manufacturer's protocol.

### Acquisition of histological images

The specimen slides were scanned with an Aperio SC2 scanner (Leica Biosystems, Wetzlar, Germany) and imaged with an Aperio eSlide Manager (Leica Biosystems). The sections were digitally imaged at 20× magnification and saved as TIFF files.

### Evaluation of immunohistochemical staining

Vascular regions (i.e., tumor areas within 100 µm of CD31-positive vessels) and avascular regions (i.e., tumor area 100 µm away from CD31-positive vessels) that stained positive for HIF1α and hyopoxyprobe-1 were analyzed using Image J software (Version 1.52a)^53^. Briefly, we took 26 color images from randomly selected vascular and avascular areas in the stained specimens at 20× magnification. The non-tumor regions and necrotic regions were removed. The color images were converted to 8-bit grayscale, and an appropriate threshold was set prior to Image J analysis.

### Evaluation of the accuracy of our system for preserving spatial information by MRI imaging (MRI)

We performed MRI at the Central Institute for Experimental Animals (Yokohama, Japan) to evaluate the accuracy of the anatomical information obtained and preserved using our method. The mice were anesthetized with 1.5% isoflurane. Respiration and rectal temperature were monitored during the measurements. MRI was performed using a 7.0 T MRI system equipped with actively shielded gradients at a maximum strength of 700 mT/m (BioSpec 70/16; Bruker BioSpin GmbH, Ettlingen, Germany) and with a 38 mm volume coil. T2-weighted images of the axial plane were acquired using a rapid acquisition method with relaxation enhancement and the following parameters: effective echo time, 40 ms; repetition time, 3500 ms; rapid acquisition with relaxation enhancement factor, 8; number of averages, 4; spatial resolution, 150 × 150 μm; slice thickness, 0.75 mm; and number of slices, 36.

### Statistical analysis

Statistical analysis was performed using SPSS Statistics version 19 (IBM, Armonk, NY, USA) based on analysis designs of our previous studies^56,57^. Statistical significance was determined using an unpaired *t*-test in the comparison of staining positive areas. For analyzing correlation between the HIF1α staining area and Hypoxyprobe-1 staining area, the variables were compared using Pearson’s correlation coefficient. A P-value of < 0.05 was considered to show statistical significance.

### Ethics approval/consent to participate statement

No data involving human patients or tissue were included and therefore our study did not require ethical approval for human rights. All animal protocols had ethical approval from the Kanagawa Cancer Center Institutional Animal Experiment Committee (Approval No. 01-07). The study is reported in accordance with ARRIVE guidelines.

## Supplementary Information

Supplementary Legends.

Supplementary Figure S1.

Supplementary Figure S2.

Supplementary Figure S3.

Supplementary Figure S4.

## Data Availability

All data generated or analyzed during this study are included in this published article and its supplementary information files. All data are available from the corresponding author upon reasonable request.
