# Supplementary material for: A practical spatial analysis method for elucidating the biological mechanisms of cancers with abdominal dissemination in vivo
Source: Sci Rep. 2022 Nov 24;12:20303. doi: 10.1038/s41598-022-24827-w (PMC9700726; doi:10.1038/s41598-022-24827-w)
Supplement: Supplementary file 1 — Supplementary Legends. [file 41598_2022_24827_MOESM1_ESM.docx]

**Supplementary information**

**SUPPLEMENTARY Figure. 1**

(a) Additional sectioning method for preserving spatial information. (b) Images of sectioned organs from nude BALB/c mice with RMG-1 ovarian cancer dissemination.

**SUPPREMENTALY Figure. 2**

Staining with RNA-ISH for retroperitoneal adipose tissue using a negative control probe (a), and anti-FABP4 probe (b and c). Scale bar: 100 µm.

**SUPPREMENTALY Figure. 3**

Comparison of measuring methods for cancer dissemination.

(a) and (b) Cancer dissemination evaluation by peritoneal cancer index (PCI) scoring and by our method. (b) HLA staining was performed to detect disseminated human cancer cell lines in the sectioned mice organs. The left-side shows the original image of the spatial sectioned tissue stained for HLA. The right-side shows a binarized image processed by Image J software. Scale bar: 5 mm.

**SUPPREMENTALY Figure. 4**

Comparison of the quality of IHC and RNA between specimens prepared under different conditions. Left, FFPE specimen processed with EDTA decalcification and gel solidification. Middle, FFPE specimen processed without EDTA decalcification and with gel solidification. Right, FFPE specimen processed without EDTA decalcification and gel solidification. (a), Perillipin1 and CD31 were stained for adipocytes and blood vessels. Scale bar: 2 mm (H&E (macro)), and 200 µm (H&E (x10), Perillipin1, and CD31). (b) DV200 results and electropherograms of RNA obtained from specimens prepared under different conditions obtained using the Agilent 2100 bioanalyzer.
