# Supplementary figures and images for: A practical spatial analysis method for elucidating the biological mechanisms of cancers with abdominal dissemination in vivo
Source: Sci Rep. 2022 Nov 24;12:20303. doi: 10.1038/s41598-022-24827-w (PMC9700726; doi:10.1038/s41598-022-24827-w)

Supplementary Fig. S1

a

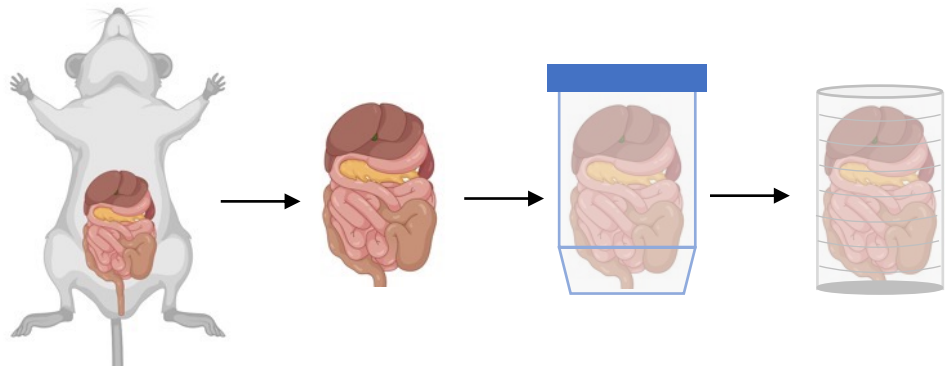

b

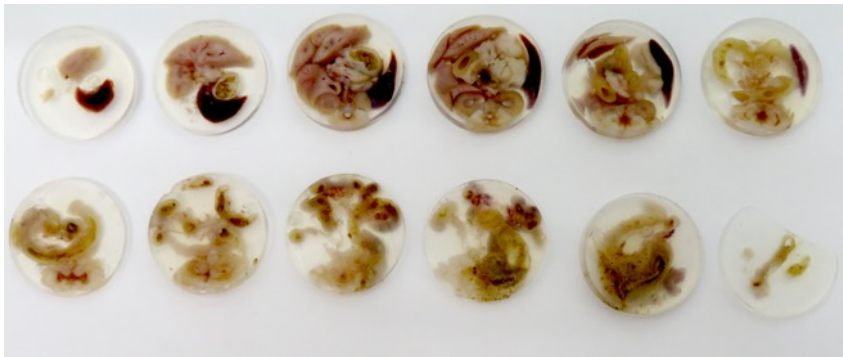

Supplement: Supplementary file 2 — Supplementary Figure S1. [file 41598_2022_24827_MOESM2_ESM.pdf]

**Supplementary Fig. S2**

a

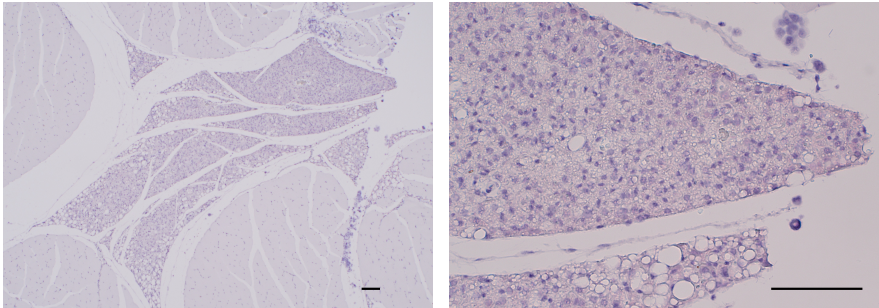

b

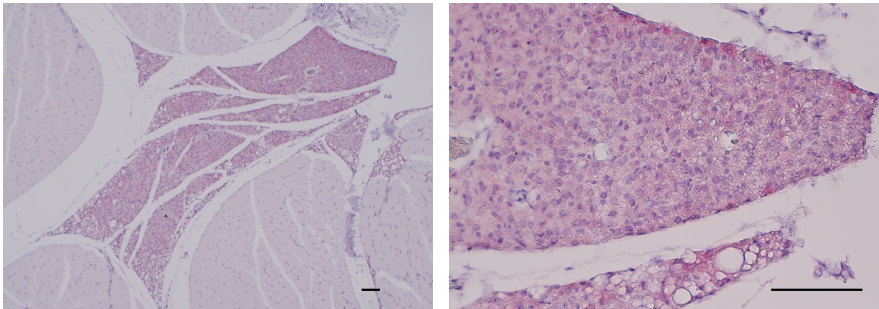

c

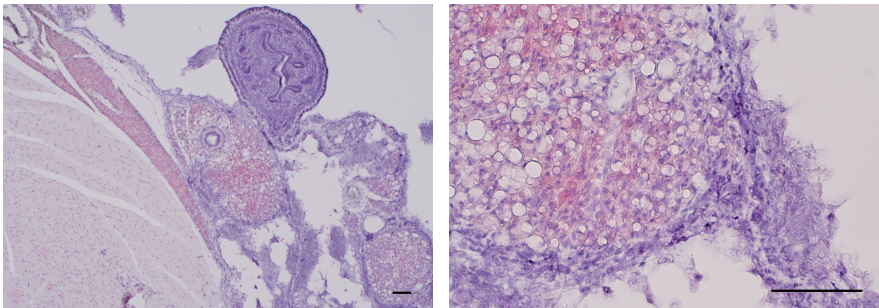

Supplement: Supplementary file 3 — Supplementary Figure S2. [file 41598_2022_24827_MOESM3_ESM.pdf]

# Supplementary Fig. S3

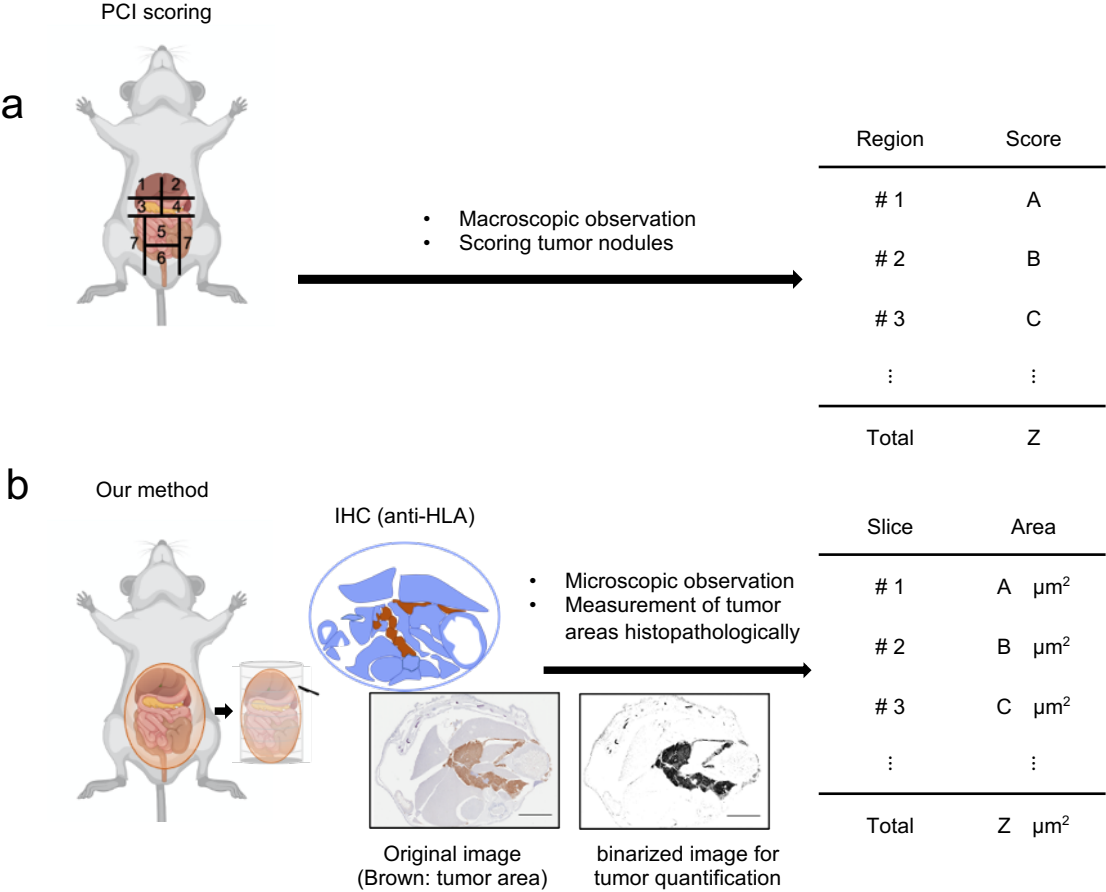

Supplement: Supplementary file 4 — Supplementary Figure S3. [file 41598_2022_24827_MOESM4_ESM.pdf]

Supplementary Fig. 4

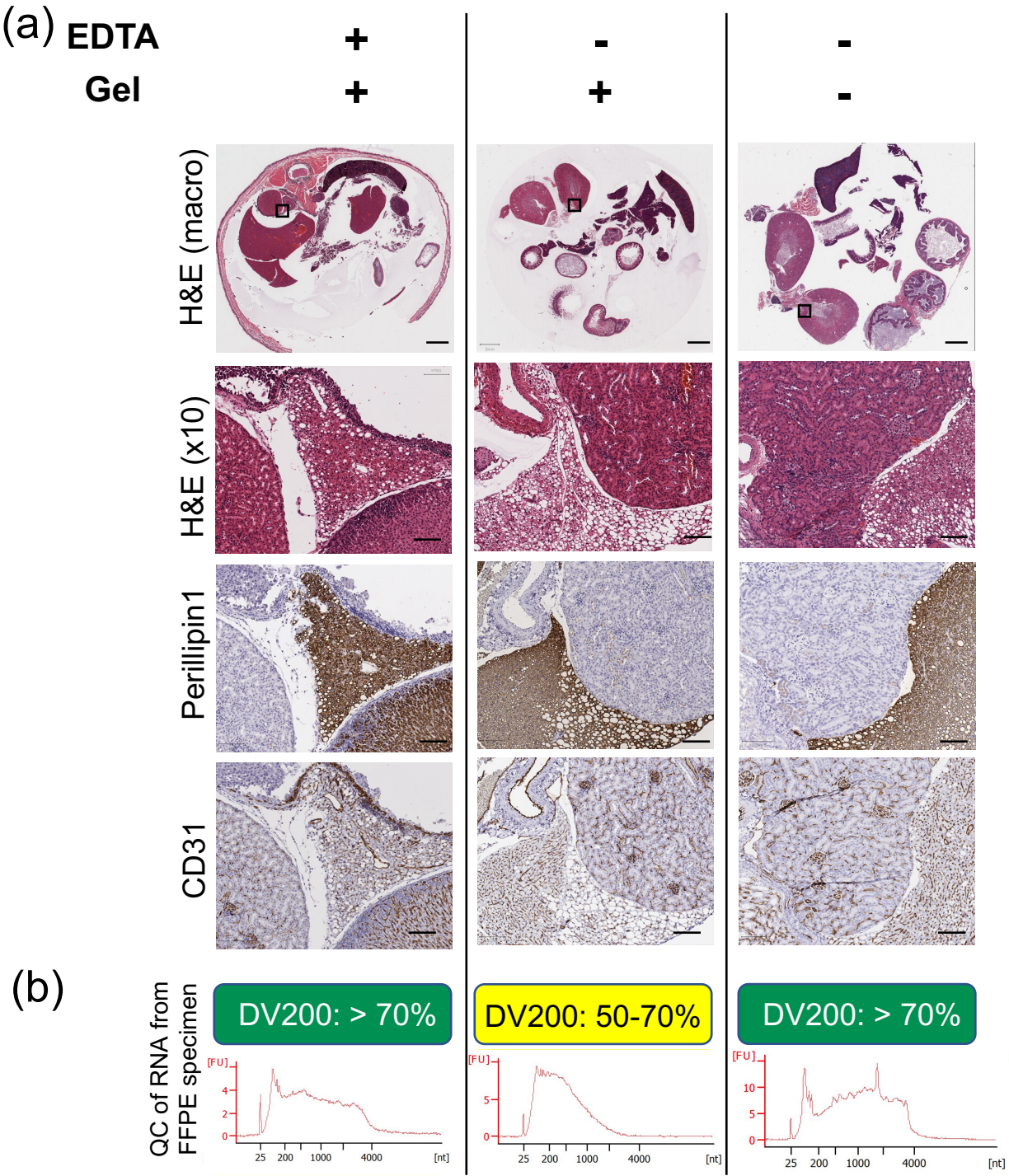

Supplement: Supplementary file 5 — Supplementary Figure S4. [file 41598_2022_24827_MOESM5_ESM.pdf]
